# Administration of Neuropsychological Tests Using Interactive Voice Response Technology in the Elderly: Validation and Limitations

**DOI:** 10.3389/fneur.2013.00107

**Published:** 2013-08-09

**Authors:** Delyana Ivanova Miller, Vincent Talbot, Michèle Gagnon, Claude Messier

**Affiliations:** ^1^School of Psychology, University of Ottawa, Ottawa, ON, Canada; ^2^TelAsk Technologies, Ottawa, ON, Canada

**Keywords:** automated telephone systems, neuropsychological evaluation, aging, working memory, e-health, computer testing

## Abstract

Interactive voice response (IVR) systems are computer programs, which interact with people to provide a number of services from business to health care. We examined the ability of an IVR system to administer and score a verbal fluency task (fruits) and the digit span forward and backward in 158 community dwelling people aged between 65 and 92 years of age (full scale IQ of 68–134). Only six participants could not complete all tasks mostly due to early technical problems in the study. Participants were also administered the Wechsler Intelligence Scale fourth edition (WAIS-IV) and Wechsler Memory Scale fourth edition subtests. The IVR system correctly recognized 90% of the fruits in the verbal fluency task and 93–95% of the number sequences in the digit span. The IVR system typically underestimated the performance of participants because of voice recognition errors. In the digit span, these errors led to the erroneous discontinuation of the test: however the correlation between IVR scoring and clinical scoring was still high (93–95%). The correlation between the IVR verbal fluency and the WAIS-IV Similarities subtest was 0.31. The correlation between the IVR digit span forward and backward and the in-person administration was 0.46. We discuss how valid and useful IVR systems are for neuropsychological testing in the elderly.

## Introduction

Interactive voice response (IVR) systems are generally described as computer systems interacting with people who, in turn, use the telephone keypad or speech to give answers to computer prompts. The use of IVR systems in psychological and neuropsychological assessment is a relatively new development.

Cognitive testing over the telephone has been used for over a decade. In the early applications, a “real” person performed both the administration and the scoring of the tests. These include the Telephone Adaptation of the Mini Mental State Examination (T3MS) (1), the Telephone Screening of Cognitive Status (TICS) (2), and its modified version TICS-m (3), used for dementia screening purposes. Other examples include the Minnesota Cognitive Acuity Screen (MCAS) for screening of dementia (4), the Telephone-Administered Cognitive Test battery (TACT) (5) and the Indiana university telephone-based assessment of neuropsychological status (6). Various studies that examined the validity of these telephone-administered tests reported good validity (7–10). The use of such tests for dementia screening may be appropriate in population studies but may not be as useful to establish more precise diagnosis such as mild cognitive impairment or sufficient to make a final diagnosis of dementia (9). Researchers have expressed concerns about the construct validity of the adapted tests and suggested that tests administered by a technology platform (WEB, computer, telephone) may measure additional and/or different cognitive abilities (11).

An example of an assisted computerized assessment is the Computer Assisted Telephone Interview (CATI) (12), which adapted the TICS-m for a computerized telephone administration. In a few instances, the computer program performs the administration and the scoring of the tests. An example of such system is the Computer Automated Dementia Screening instrument (IVR-ADS) (13). A number of factors explain the proliferation of IVR systems. The telephone is still the most widely adopted technology. In 2008 in Canada, 99.1% of households own and use a telephone (home or cellular) compared to other communication devices such as the computer (79.4%). Within the older population, 98.9% own and use a telephone compared to 33.1% for home computers, and only 26.6% of older adults access Internet at home (14). Another obvious advantage is that IVR systems are less costly than live operators (15) and are continuously available. Finally, IVR systems give callers the impression of increased privacy compared to live person interaction particularly when communicating sensitive information (16, 17).

In the present experiment, we report on the development and preliminary validation of IVR algorithms, which administer and score in real time three commonly used neuropsychological tests using voice as the sole interaction mode. The goal of the present study was to evaluate the feasibility of the IVR application in older adults. In the course of this study, participants were also administered the Wechsler Intelligence Scale fourth edition (WAIS-IV) and Wechsler Memory Scale fourth edition (WMS-IV) so an estimate of the validity of the IVR versions of the verbal fluency task (naming fruits), and the Digit Span Forward and Backward could also be examined.

## Materials and Methods

### Participants

The present study and all corresponding documentation was reviewed and approved by the Research Ethics Board of the University of Ottawa. One hundred and fifty-eight (105 females: 66.5%) community dwelling people between 65 and 92 years of age (*M* = 72.97 years, SD = 6.23) were recruited from diverse socio-economic backgrounds, using advertisements in two free magazines for seniors and flyers, in community centers and subsidized housing buildings. Participants’ education ranged from 7 to 21 years (*M* = 13.85 years, SD = 2.79); 2% of participants had grade 8 or less, 14% had between grade 9 and grade 11, 34% had a high school diploma, 18% had some college or university, and 32% had a bachelor’s, graduate, or professional degree. Full scale IQ ranged from 68 to 134. The only exclusion criteria were age younger than 65 and lack of proficiency in English. Participants gave informed consent and were compensated $100. Self-reported demographic and health information was gathered using a health questionnaire. Eighty-eighth percent were Caucasian, 9.5% reported being diabetic, 0.6% reported having had a hemorrhagic stroke, 1.3% had been treated for a brain tumor, 1.3% reported another unspecified brain disease, and 0.6% had chronic hepatitis, 2.5% reported currently seeing a psychiatrist, and 10.8% were currently being treated for depression. Sixty percent of the sample reported experiencing memory problems.

### Measures

We used the Wechsler Intelligence Scale third edition (WAIS-III) version of Digit Span (18) for IVR administration and the WAIS-IV version (19) for the in-person administration in order to reduce practice effects. In the WAIS-IV, the Digit Span task was revised to include an additional section to the subtest, the Sequencing Digit Span. Some of the numbers in the Digit Span Forward and Backward were changed in order to eliminate similar sounding stimuli. The task has been found to be sensitive to age related cognitive decline and a number of neurological disorders (20).

The Animal Naming Task is a semantic fluency task. The task asks the examinee to name as many animals as the person can think of in 1 min and measures person’s ability to rapidly generate words in response to a semantic cue (animals) (20). Several alternate forms of the task have been developed and normed: naming vegetables, fruits, foods, and clothing (21–23). Semantic fluency has been shown to significantly decline as people age (22, 24).

The Wechsler Intelligence Scale fourth edition ten core subtests: Block Design, Similarities, Digit Span, Matrix Reasoning, Vocabulary, Arithmetic, Symbol Search, Visual Puzzles, Information, and Coding were administered by trained psychometricians following the standard clinical administration described in the test documentation. The WAIS-IV, older adult battery (WMS-IV, older adults) consists of Logical Memory immediate and delayed recall, Visual Reproduction immediate and delayed recall, Verbal Pairs immediate and delayed recall, and the Symbol Span subtests.

The word recognition engine was the Nuance Open Speech Recognizer version 3.0.3. There was no prior training of the system to optimize speech recognition of individual participants. A female professional voice talent recorded all the instructions and statements produced by the computer. The algorithms for the IVR tasks were developed by TelAsk technologies and two versions of the automated system were piloted using focus groups (25). The subsequent versions of the IVR tasks were improved using the feedback from participants taking part in the focus groups. The final versions were used in this study. In addition to the three neuropsychological tests, the system allowed for the option of adjusting the volume and the speed of the conversation at the beginning of the interaction as well as for repetition of all instructions. There were five levels for the volume and the default was set at the medium volume 3. The speed adjustment had three levels and the default was set at 2.35 words/s. The two other levels were slower (approximately 1.78 and 1.6 words/s). These two slower levels were not digitally modified: rather the voice talent changed her speech rate in these recordings. All the interactions between the computer and the participants were recorded. The three tasks were scored by the IVR system and by a clinician (using the recordings) in order to examine the reliability of the system’s scoring.

The verbal fluency task was presented first; the examinees were instructed to name as many fruits as they could think of. They were also told that they had 1 min to complete the task. The Digit Span Forward task was presented second and the Digit Span Backward was presented last. For the two Digit Span tasks, we used the standard administration instructions from the WAIS-III manual. We also introduced a beep in the IVR version, which was the cue indicating that the string of numbers had been presented and participants needed to generate a response. This cue was introduced after pilot administrations revealed that many participants were waiting for additional digits and hesitant to start responding. We realized that the lack of a verbal (or non-verbal) cue for the last number, which would have been present in the in-person administration, led to uncertainty as to when the string of numbers ended on each trial.

Upon arriving at the memory laboratory at the University of Ottawa, self-reported information on participants’ health and memory status was obtained using the health questionnaire. Next, participants completed the IVR tasks over the phone. Participants were not allowed to take notes during the interaction. In addition, all participants were administered the WAIS-IV and WMS-IV (older adult) batteries.

A touch-tone phone (MITEL 5212) was used to call the IVR systems. A Sony MP3 IC recorder (ICD-UX7 1F/UX81F) was attached to the phone line and Sony stereo headphones (MDR-XD200) was attached to the recorder. Thus, the headphones allowed the examiners to listen to participants’ interaction with the IVR system as the communication was unfolding.

## Results

Out of the 158 participants involved in the study, 152 completed all three tasks, one participant failed to complete the Digit Span Forward task and six participants completed only the verbal fluency task. The seven participants who completed only one or two tasks had difficulty interacting with the system, which caused the system to discontinue the conversation. Eleven percent of participants used the adjustment of the volume option; with six percent of them using the option to increase the volume and five percent of them used it to decrease the volume of the conversation. Fifteen percent of participants used the slowing down the speed of the conversation option. Eleven percent of participants asked the system to repeat the instructions for the verbal fluency task and nine percent of participants did so for the digit span forward task.

The procedure and data analysis of this experiment were designed to answer three main questions. The first one was to determine if an IVR system could independently administer some simple neuropsychological tests and what were the problems associated with this administration. The second question was to determine if an IVR system could score the responses provided by the participants with accuracy and, if not, what were the technical hurdles that reduced accuracy. The final question, the most interesting to professionals that administer these tests, was whether neuropsychological tests administered over the phone using an IVR system provide comparable results to in-person administration with the caveat that for two of these tests (digit span forward and backward), the in-person administration followed the IVR administration (there was no counter-balancing of the order). Let’s turn now to each of the tests and a detailed analysis of the achievements and limits of each IVR test.

### Verbal fluency task

#### Administration

All participants completed the verbal IVR fluency task and no problems occurred during the administration. The fluency task involved little interaction between the IVR system and the participant beyond adjusting the speed of delivery and sound level or requesting repetition of instructions.

#### Scoring

The correlation between the IVR verbal fluency task scored by the IVR system and scored by a clinician was 0.89, *p* < 0.01. The mean number of words scored by a clinician was higher (*M* = 10.68, SD = 4.28) compared to the scoring by the system (*M* = 9.68, SD = 3.66). The system was able to correctly recognize 90% of the fruit that participants named (in total participants named 1716 words, and the system identified 1539 words). Participants named 45 exotic fruit that were not part of the initial list entered into the system (e.g., breadfruit, cactus fruit, paw paws fruit, dragon fruit, etc.). The system failed to recognize 132 fruits (this number excludes the fruit that were not on the list: an error rate of 8%). Qualitative examination of the data revealed that the system’s failure to correctly recognize some of the words was due to language issues such as non-English accent, poor pronunciation, and verbal behaviors such as coughing, speaking inaudibly (very quietly), saying “umm,” mumbling, and speaking in full sentences to the system.

The clinician and IVR scoring of the test were identical in only 41.1% of cases (see Table 1). In 5.7% of cases, the system gave one point higher score indicating that some of the repetitions or non-fruit words were identified as a correct response by the system. In the majority of cases (53.2%), the system gave a lower score than the one assigned by a clinician. For 27% (*N* = 25) of people who had discrepancy in scores between clinician and system scoring the difference was due to fruits that were not part of the list that was entered into the system. Eleven percent (*N* = 10) of the people with discrepant scores had foreign accents and 10% (*N* = 9) had pronunciation difficulties. For the rest of participants (5.2%), the discrepancy was likely due to verbal behaviors described earlier.

**Table 1:** **Number of people with a discrepancy between clinician scoring and system scoring**.

Discrepancy*	Frequency	Percent
−1.00	9	5.7
0.00	65	41.1
1.00	32	20.3
2.00	27	17.1
3.00	13	8.2
4.00	5	3.2
5.00	4	2.5
7.00	2	1.3
8.00	1	0.6
Total	158	100.0

*Positive numbers represent higher scores given by the clinician.

#### Comparison with in-person administration

Unfortunately, at the time of the design of the study, we did not include a variation of the verbal fluency task (e.g., animals). However, we compared participants’ performance on the IVR Verbal Fluency task to their performance on the Similarities subtest of the WAIS-IV administered in-person. The Similarities subtest is the closest subtest in terms of cognitive functions being assessed (abstract verbal abilities). For all participants who completed the IVR Verbal Fluency task, the correlation between their IVR generated score and the Similarities subtest was *r* = 0.32, *p* < 0.01. The correlation between the clinician scoring of the Verbal Fluency task and the Similarities subtest was *r* = 0.31, *p* < 0.01. When we excluded the participants for whom the system and clinician scoring were discrepant, the correlations between the IVR Verbal Fluency task and the WAIS verbal subtest were the Similarities subtest was 0.47, *p* < 0.01.

### Digit span forward

#### Administration

For both the digit span forward and backward, participants had no problems completing the IVR tests. The administration of the IVR tests depended on the accurate recognition by the IVR system of each response to determine whether to continue with a number presentation in the same level or a number presentation for the next higher level. In the case where the IVR system did not recognize both sets in one level or when participants missed the first set of numbers and the IVR system did not recognize the second set, the test was incorrectly terminated.

#### Scoring

In the following discussion, scores refer to the number of digit sequences correctly repeated. The correlation between the Digit Span Forward task (adapted from the WAIS-III) scored by the IVR system and scored by a clinician was 0.95, *p* < 0.01. The mean number of digit sequences correctly repeated was (*M* = 8.80, SD = 3.10) for the clinician scoring and (*M* = 8.34, SD = 3.13) for the system scoring. The system was able to correctly recognize 95% of the digit sequences repeated by participants (participants named 1337 sequences and the system correctly identified 1268 of them). The system and clinician scoring of the IVR Digit Span Forward task was identical for 63.8% of the cases (see Table 2). In 3.3% of cases, the system gave a higher score than the one assigned by the clinician. In the rest of the cases (32.9%), the system gave lower score than the one assigned by the clinician. The most common causes of failure on the part of the IVR to recognize a response are presented in Table 3. In many instances, the system by virtue of recognizing one out of two strings of number of the same length allowed the participant to continue to the next level. However, the system discontinued the task for 12.5% (*N* = 19) of participants earlier than it should have. One third of participants for whom the task was discontinued early (*N* = 7) had foreign accent or difficulties with pronunciation. In addition, a number of people had a combination of issues while completing the task that may have caused verbal recognition difficulties and led to discrepancy in scoring between clinician and the IVR system (see Table 4).

**Table 2:** **Number of people with a discrepancy between System Scoring and Clinician Scoring IVR Digit Span Forward**.

Discrepancy*	Frequency	Percent
−4.00	1	0.7
−1.00	4	2.6
0.00	97	63.8
1.00	30	19.7
2.00	14	9.2
3.00	5	3.3
4.00	1	0.7
Total	152	100.0

*Positive numbers represent higher scores given by the clinician.

**Table 3:** **Issues causing verbal recognition problems by the system (Digit Span Forward task)**.

Problem	*N*	Percentage
Overall discrepancy between scoring	55	36.2
Participants correcting their responses	19	12.5
System did not allow for enough time to respond	6	3.9
System did not “hear” responses	14	9.2
Participants speaking in low voice	9	5.9
Foreign accent or bad pronunciation	21	13.8
Participant did not wait for the beep	20	13.2
Other verbal problems (e.g., coughing and “Ummm”)	15	9.9
Speaking in sentences	13	8.6

**Table 4:** **Number of issues encountered during the IVR Digit Span Forward task**.

Number of issues	Frequency	Percent
0.00	98	64.5
1.00	17	11.2
2.00	15	9.9
3.00	19	12.5
4.00	2	1.3
5.00	1	0.7
Total	152	100.0

#### Comparison with in-person administration

For all participants who completed the IVR Digit Span Forward, the correlation between their IVR scores and the in-person administration of the task was *r* = 0.46, *p* < 0.01. The correlation between clinician scored IVR Digit Span Forward and the in-person administration of the task was *r* = 0.48, *p* < 0.01. For the 63.8% of participants for whom the IVR and clinician scoring of the Digit Span Forward task was identical, the correlation between their IVR Digit Span Forward (adapted from the WAIS-III) and the in-person administered Digit Span Forward (WAIS-IV) was *r* = 0.41, *p* < 0.01. The mean number of digit sequences repeated correctly in the in-person administration of the Digit Span Forward was: *M* = 9.58, SD = 2.30, which was significantly higher than the IVR Digit Span Forward score (*M* = 8.80, SD = 3.10) as indicated by paired samples *t*-test [*t*(96) = 3.99, *p* < 0.01]. One contributor of this difference was the early termination of the task for some participants.

### Digit span backward

#### Scoring

The correlation between the Digit Span Backward (adapted from the WAIS-III) scored by a clinician and by the IVR system was *r* = 0.94, *p* < 0.01. Once again, the mean number sequences repeated backwards was higher when scored by a clinician (*M* = 6.07, SD = 2.33) compared to the system scoring (*M* = 5.63, SD = 2.18). The system was able to recognize 93% of the digit sequences repeated backward by participants (participants repeated 928 sequences and the system recognized 861 of them). The system and clinician scoring of the IVR Digit Span Backward task was identical in 68% of cases (see Table 5). There was only one participant to whom the system assigned a higher score than the one assigned by the clinician. For the rest of participants (31.3%) the system gave a lower score. Examination of the audio recordings revealed the same issues as the one described for the Digit Span Forward task (see Table 6). In addition, for 35% of participants who had discrepancy in scoring between a clinician and the system, the presentation of the Digit Span Backward task was discontinued early. Only two participants for whom the task was discontinued early had accent and pronunciation difficulties. A number of people had a combination of issues while interacting with the system that likely led to recognition problems (see Table 7).

**Table 5:** **Number of people with a discrepancy between System Scoring and Clinician Scoring for the IVR Digit Span Backward**.

Discrepancy	Frequency*	Percent
−2.00	1	0.7
0.00	104	68.0
1.00	31	20.3
2.00	11	7.2
3.00	6	3.9
Total	153	100.0

*Positive numbers represent higher scores given by the clinician.

**Table 6:** **Issues causing verbal recognition problems by the system (Digit Span Backward task)**.

Problem	*N*	Percentage
Overall discrepancy between scoring	49	32
Participants correcting their responses	15	9.8
System did not allow for enough time to respond	26	17
System did not “hear” responses	3	2
Participants speaking in low voice	6	3.9
Foreign accent or bad pronunciation	12	7.8
Participant did not wait for the beep	14	9.2
Other verbal problems (e.g., coughing and Ummm)	10	6.5
Speaking in sentences	10	6.5

**Table 7:** **Number of issues encountered during the IVR Digit Span Backward task**.

Number of issues	Frequency	Percent
0.00	105	68.6
1.00	11	7.2
2.00	19	12.4
3.00	10	6.5
4.00	6	3.9
5.00	2	1.3
Total	153	100.0

#### Comparison with in-person administration

For all participants who completed the task, the correlation between the IVR generated scores for the Digit Span Backward and the in-person administration of the task was *r* = 0.46, *p* < 0.01. The correlation between clinician generated scores and the in-person administration of the task was *r* = 0.50, *p* < 0.01. For the 68% of participants for whom the IVR and clinician scoring of the Digit Span Backward task was identical, the correlation between their IVR Digit Span Backward (adapted from the WAIS-III) and the in-person administered Digit Span Backward (WAIS-IV) was *r* = 0.52, *p* < 0.01. The mean number of digit sequences repeated correctly in the in-person administration of the Digit Span Backward was (*M* = 8.59, SD = 2.19), which was once again significantly higher than that mean number of digit sequences obtained in the IVR version (*M* = 6.07, SD = 2.33) as indicated by paired samples *t*-test [*t*(103) = 13.98, *p* < 0.01].

### Results for the three IVR tasks combined

We also examined how many people had discrepancy in scoring on one or more of the IVR tasks. Frequency analyses revealed that only 20.9% of participants had identical clinician and IVR generated scores for all three tasks, 39.9% of participants had discrepancies in scoring for one of the tasks, 30.7% had differences in scoring for two of the tasks and 8.5% of people had discrepant scores on all three tasks. We conducted one-way ANOVAs in order to examine if the different groups (people who had discrepancies on none, one, two, or all three tasks) were different in terms of their age, level of education, verbal and perceptual abilities, full scale IQ, verbal and visual memory, and immediate and delayed memory. The ANOVAs were not significant for any of these variables. In order to determine the cognitive functions that the three IVR tasks measured, the scores obtained on the IVR tasks were correlated with the WAIS-IV subtests results but we included only the participants for which the IVR system and the clinician scores were the same (see Table 8).

**Table 8:** **Correlations between the IVR tests (for the people for whom clinician and IVR generated scores were identical) and the in-person administered WAIS-IV battery**.

	IVR Flu.	Similar.	IVR DSF	DSF	IVR DSB	DSB
Block D.	0.096	0.373**	−0.188	0.104	0.196*	0.165*
Similarities	0.470**		0.118	0.160*	0.206*	0.160*
Digit forward	−0.121	0.160*	0.407**		0.322**	0.424**
Digit backward	0.015	0.160*	0.202*	0.424**	0.524**	
Matrix	0.232	0.438**	−0.096	0.105	0.074	0.228**
Vocabulary	0.532**	0.631**	0.183	0.261**	0.232*	0.253**
Arithmetic	−0.071	0.348**	0.028	0.287**	0.452**	0.333**
Symbol S.	−0.019	0.354**	0.056	0.176*	0.315**	0.216**
Visual Puz.	0.134	0.325**	0.009	0.133	0.118	0.241**
Information	0.422**	0.473**	0.207*	0.068	0.046	0.104
Coding	0.014	0.368**	0.081	0.163	0.311**	0.161

*Significant at the 0.05 level.

**Significant at the 0.01 level.

The IVR verbal fluency task was significantly correlated with all WAIS-IV verbal tasks: Similarities, Vocabulary, and Information. The Digit Span Forward was significantly correlated with the WAIS-IV Digit Span Forward, Digit Span Backward, and the Information subtests. The Digit Span Backward correlated with a number of tasks from the WAIS-IV measuring not only working memory but also verbal abilities and processing speed.

In order to compare how well the IVR-administered Digit Span Forward and backward tasks compare to the in-person administration of the tasks we also generated a correlation matrix of participants’ scores on the 10 core subtests of the WAIS-IV (see Table 8). The most striking differences were the higher correlation between the Digit Span Forward task and the Digit Span Backward task for the in-person administration compared to the correlation of the IVR administration (InP: *r* = 0.424, *p* < 0.01; IVR: *r* = 0.202, *p* < 0.05), higher correlation of Digit Span Forward with the Arithmetic task for the in-person administration (InP: *r* = 0.287, *p* < 0.01; IVR: *r* = 0.028, ns), and higher correlation for the in-person administration of Digit Span Forward and the Vocabulary task (InP: *r* = 0.261, *p* < 0.01; IVR: *r* = 0.183, ns). The correlation between the in-person administration of the Digit Span Backward task were higher with the Digit Span Forward (InP: *r* = 0.424, *p* < 0.01; IVR: *r* = 0.322, *p* < 0.01) and higher correlation with the Matrix subtest for the in-person administration of the Digit Span Backward (InP: *r* = 0.228, *p* < 0.01; IVR: *r* = 0.074, ns). In secondary analyses (data not shown), we examined whether the participants who slowed down the speed of the IVR conversation, adjusted the volume or repeated the instructions for the tasks had lower overall scores on the in-person administered cognitive batteries. No significant differences were found.

Principal component analyses with a varimax rotation was used on the data, and the variables included the three IVR-administered tests (Verbal fluency, Digit Span Forward, and Digit Span Backward) and the raw scores of the 10 core subtests of the WAIS-IV. Eigenvalues indicated that the first three factors explained 32.9, 13.7, and 9.7% of the variance respectively, for a total of 56% of the variance, which was also the case after varimax rotation was performed on the data. Thus, a three-factor solution was retained (see Table 9). The IVR Verbal Fluency task loaded on the second factor 0.573, together with all verbal tasks of the WAIS-IV (Similarities −0.718, Vocabulary −0.771, and Information −0.778) providing support for the strong verbal component of the task. The IVR Digit Span Forward and Backward loaded on the third factor (0.697 and 0.684, respectively) together with the Digit Span Forward and Backward from the WAIS-IV (0.739 and 0.748, respectively). It was not surprising that the Forward and Backward versions of the task clustered together as in the WAIS these two tasks are part one subtest under the working memory index. The finding that the IVR Digit Span tasks loaded on the same factor as the WAIS-IV Digit Span tasks indicates that the tasks are tapping into the same core latent abilities.

**Table 9:** **Principal component analyses with a varimax rotation for IVR tasks and the raw scores of the 10 core subtests of the WAIS-IV**.

**VARIMAX ROTATED MATRIX**			

	Components		
	1	2	3
Number of words without repetitions
and errors	0.036	0.573	0.186
IVR-COG digit span forward	−0.185	0.268	0.697
IVR-COG digit span backward	0.280	−0.036	0.684
Raw score for block design	0.715	0.248	−0.048
Raw score for similarities	0.334	0.718	0.096
Raw score for digit span forward	0.040	0.133	0.739
Raw score for digit span backward	0.214	0.009	0.748
Raw score for matrix reasoning	0.627	0.405	−0.032
Raw score for vocabulary	0.277	0.771	0.203
Raw score for arithmetic	0.471	0.220	0.371
Raw score for symbol search	0.718	0.062	0.160
Raw score for visual puzzles	0.705	0.141	0.127
Raw score for information	0.210	0.778	−0.075
Raw score for coding	0.746	0.150	0.122

## Discussion

The majority of participants who were administered the IVR tasks were able to complete all three tasks and only 4% of the participants had difficulties that caused them to complete only one or two of the tasks. Thus, it appears that the IVR system was easy to use and instructions were presented in a way that was understood by participants. Because there were no exclusion criteria except age, language proficiency, and self-exclusion from the study, the sample included people with lower IQ and a number of people with significant memory problems that would fit the description of mild cognitive impairment. Although we made no effort to identify people with mild dementia, anecdotal evidence indicated that a few of our participants had early dementia symptoms but were able to complete all three tests.

The correlation between the IVR scoring of the verbal fluency task and the clinician scoring was high (*r* = 0.89), and the verbal recognition error of the system was low (8% recognition error). However perfect agreement between the automated scoring and the clinician scoring was found in only 41.1% of cases. In 53.2% of cases, the system failed to recognize responses that were part of the initial list and assigned lower scores to participants. Improvements in the speech recognition engines and programing the IVR system will be needed to recognize behaviors such as low voice volume and speaking in sentences. The lack of high agreement between clinician and computer scoring remains the greatest obstacle to the use of IVR systems in the clinic.

The mean number of fruits named by participants also tended to be low (10.68) compared to other studies using verbal fluency tasks (animals, fruits, items at the supermarket) with similar samples: in these studies, the range of items produced ranged from 9 to 23 (21, 26, 27). This could be due to the fact that these studies had more stringent exclusion criteria or different recruitment strategies that may have led to healthier and/or more educated participants. Another but less likely possibility is that the computer interaction somehow inhibited participants’ responses or distracted them from the main task.

One important limitation of our study was the lack of an in-person administration of an alternate version of the verbal fluency task. Thus, we compared participants’ performance on the IVR verbal fluency task to their performance on the Similarities subtest of the WAIS-IV, which was the closest test in terms of cognitive abilities required (executive function and verbal abilities). There was only a modest relationship between the two variables (*r* = 0.47). However, the verbal fluency task correlated significantly with all verbal tests of the WAIS-IV providing support for the strong verbal component of the task.

The Digit Span Forward and Digit Span Backward administered by the system had very high correlations between the system and the clinician scoring (*r* = 0.89 and *r* = 0.95, respectively) and low verbal recognition error rate (5 and 7%, respectively); however, the scoring between the clinician and the system matched in only 63.8% of cases for the Digit Span Forward task and in only 68% of cases for the Digit Span Backward task. This was due to the problems noted above for the verbal fluency task.

The correlations between the in-person administration of the Digit Span Forward and Backward and the IVR digit span tasks for the participants for which the system and the clinician scoring was identical were also modest (*r* = 0.41 for the Digit Span Forward, and *r* = 0.52 for the Digit Span Backward). The modest correlations indicate that a number of participants who obtained high scores on the IVR tasks received a lower score on the in-person administration and vice versa (the scatterplot was oval.) We also noted that the mean performance of participants as a group was higher for the in-person administration compared to the IVR tasks. These results are consistent with another investigation, which reported higher mean scores for the in-person administration of tests compared to telephone administration (even when the administration over the phone is accomplished by a person) (28). In addition, Thompson et al. (28) reported a number of low and moderate correlations between some of the tests for the in-person and telephone administration. Similarly, another study reported moderate correlation between the telephone and in-person administration of verbal learning tasks during a long delay condition (6). However, in Unverzagt study, the correlation between the Digit Span total score for telephone and in-person administration of the test was high (*r* = 0.82). The moderate correlation of the Digit Span scores in our study was somewhat surprising given that previous studies have reported comparable scores and considering that although the IVR Digit Span tasks were adapted from the WAIS-III and the in-person Digit Span tasks was from the WAIS-IV, they are essentially the same tasks with very similar administration and scoring. It is possible that some practice effects were in play for the in-person administration of the task, since the IVR was administered before the WAIS-IV. Thus, participants had some familiarity with the Digit Span Forward and Backward during the in-person administration. It is also possible that the IVR tasks are more demanding and require higher concentration because of the lack of non-verbal cues present to mark the end of the presentation of the string of numbers. These clues were absent in the IVR presentation, and to compensate for that we introduced a beep at the end of each string. However, the beep may have also introduced a distraction and may have interfered with participants’ attention and concentration.

Lastly, our findings regarding the differences in correlations between the in-person administered Wechsler subtests and IVR-administered Digit Span tasks suggest that the different mode of administration of the two tasks may change what the tests measures. For example, the IVR tasks may be tapping on more than just ability to hold numbers in mind and repeat them or hold information in mind manipulate it and generate a response.

In summary, the clinician and the IVR system scoring were highly correlated but we uncovered a number of issues related to both the administration and the scoring of the tests. Test results of the IVR administration of the tasks were not entirely comparable to the in-person administration. Some problems were obvious but there are some indications that IVR administration introduced new variables in known tests. Voice recognition software has significantly improved since our study was performed and the IVR software could take advantage of these improvements to control the administration and scoring issues we reported. However, we agree with the position expressed by Bauer (11) cautioning that tests adapted for computer administration need to be validated and normed separately because the adaptation of the tests to a telephone interview may change them significantly.

The future of IVR neuropsychological testing as a clinical tool will depend on the improvement of voice recognition engines, and of algorithms to deal with non-verbal utterances, foreign accents, and detection of sentence. The other major challenge is to norm IVR-administered tests because they do not appear to be exactly equivalent to face-to-face administration. In our experiment, IVR administration appeared to lead to lower performance. Finally, IVR tests when finally developed will remain limited to tests using verbal material and, as such, will complement face-to-face administration to increase productivity. However the use of IVR tasks in epidemiological research is less constrained and will likely be the first application of IVR-administered and scored neuropsychological tasks.

## Conflict of Interest Statement

This study was funded by the Natural Sciences and Engineering Council of Canada and by TelAsk Technologies. Vincent Talbot is a paid consultant for TelAsk.
